# Cerebrovascular Reactivity at Rest and Its Association With Cognitive Function in People With Genetic Frontotemporal Dementia

**DOI:** 10.1212/WNL.0000000000213677

**Published:** 2025-09-04

**Authors:** Ivana Kirilova Kancheva, Arabella Bouzigues, Lucy Louise Russell, Phoebe H. Foster, Eve Ferry-Bolder, John C. Van Swieten, Lize Corrine Jiskoot, Harro Seelaar, Raquel Sánchez-Valle, Robert Laforce, Caroline Graff, Daniela Galimberti, Rik Vandenberghe, Alexandre de Mendonça, Pietro Tiraboschi, Isabel Santana, Alexander Gerhard, Johannes Levin, Sandro Sorbi, Markus Otto, Simon Ducharme, Christopher Butler, Isabelle Le Ber, Elizabeth Finger, Maria Carmela Tartaglia, Mario Masellis, Matthis Synofzik, Fermin Moreno, Barbara Borroni, Jonathan Daniel Rohrer, Louise van der Weerd, James B. Rowe, Kamen Tsvetanov, for the GENFI Consortium

**Affiliations:** 1 Department of Radiology, Leiden University Medical Center, the Netherlands;; 2 Department of Clinical Neurosciences and Cambridge University Hospitals NHS Trust, University of Cambridge, United Kingdom;; 3 Dementia Research Center, Department of Neurodegenerative Disease, UCL Queen Square Institute of Neurology, London, United Kingdom;; 4 Department of Neurology, Erasmus Medical Center, Rotterdam, the Netherlands;; 5 Alzheimer's Disease and Other Cognitive Disorders Unit, Neurology Service, Hospital Clínic, Institut d'Investigacións Biomèdiques August Pi I Sunyer, University of Barcelona, Spain;; 6 Clinique Interdisciplinaire de Mémoire, Département des Sciences Neurologiques, CHU de Québec, and Faculté de Médecine, Université Laval, Canada;; 7 Department of Neurobiology, Care Sciences and Society, Center for Alzheimer Research, Division of Neurogeriatrics, Bioclinicum, Karolinska Institutet, Solna, Sweden;; 8 Unit for Hereditary Dementias, Theme Inflammation and Agеing, Karolinska University Hospital, Solna, Sweden;; 9 Fondazione Ca' Granda, IRCCS Ospedale Policlinico, Milan, Italy;; 10 University of Milan, Centro Dino Ferrari, Italy;; 11 Laboratory for Cognitive Neurology, Department of Neurosciences, KU Leuven, Belgium;; 12 Neurology Service, University Hospitals Leuven, Belgium;; 13 Leuven Brain Institute, KU Leuven, Belgium;; 14 Faculty of Medicine, University of Lisbon, Portugal;; 15 Fondazione IRCCS Istituto Neurologico Carlo Besta, Milano, Italy;; 16 University Hospital of Coimbra (HUC), Neurology Service, Faculty of Medicine, University of Coimbra, Portugal;; 17 Center for Neuroscience and Cell Biology, Faculty of Medicine, University of Coimbra, Portugal;; 18 Division of Psychology Communication and Human Neuroscience, Wolfson Molecular Imaging Center, University of Manchester, United Kingdom;; 19 Department of Nuclear Medicine, Center for Translational Neuro- and Behavioural Sciences, University Medicine Essen, Germany;; 20 Department of Geriatric Medicine, Klinikum Hochsauerland, Arnsberg, Germany;; 21 Department of Neurology, Ludwig-Maximilians Universität München, Germany;; 22 German Center for Neurodegenerative Diseases (DZNE), Munich, Germany;; 23 Munich Cluster of Systems Neurology (SyNergy), Germany;; 24 Department of Neurofarba, University of Florence, Italy;; 25 IRCCS Fondazione Don Carlo Gnocchi, Florence, Italy;; 26 Department of Neurology, University of Ulm, Germany;; 27 Department of Psychiatry, McGill University Health Center, McGill University, Montreal, Québec, Canada;; 28 McConnell Brain Imaging Center, Montreal Neurological Institute, McGill University, Québec, Canada;; 29 Nuffield Department of Clinical Neurosciences, Medical Sciences Division, University of Oxford, United Kingdom;; 30 Department of Brain Sciences, Imperial College London, United Kingdom;; 31 Sorbonne Université, Paris Brain Institute – Institut du Cerveau – ICM, Inserm U1127, CNRS UMR 7225, AP-HP - Hôpital Pitié-Salpêtrière, France;; 32 Centre de référence des démences rares ou précoces, IM2A, Département de Neurologie, AP-HP - Hôpital Pitié-Salpêtrière, Paris, France;; 33 Département de Neurologie, AP-HP - Hôpital Pitié-Salpêtrière, Paris, France;; 34 Department of Clinical Neurological Sciences, University of Western Ontario, London, Canada;; 35 Tanz Center for Research in Neurodegenerative Diseases, University of Toronto, Ontario, Canada;; 36 Sunnybrook Health Sciences Center, Sunnybrook Research Institute, University of Toronto, Ontario, Canada;; 37 Department of Neurodegenerative Diseases, Hertie-Institute for Clinical Brain Research and Center of Neurology, University of Tübingen, Germany;; 38 Center for Neurodegenerative Diseases (DZNE), Tübingen, Germany;; 39 Cognitive Disorders Unit, Department of Neurology, Donostia Universitary Hospital, San Sebastian, Spain;; 40 Neuroscience Area, Biodonostia Health Research Institute, San Sebastian, Gipuzkoa, Spain;; 41 Department of Clinical and Experimental Sciences, University of Brescia, Italy;; 42 Molecular Markers Laboratory, IRCCS Istituto Centro San Giovanni di Dio Fatebenefratelli, Brescia, Italy;; 43 Department of Human Genetics, Leiden University Medical Center, the Netherlands;; 44 MRC Cognition and Brain Science Unit, University of Cambridge, United Kingdom; and; 45 Department of Psychology, University of Cambridge, United Kingdom.

## Abstract

**Background and Objectives:**

Cerebrovascular reactivity (CVR) is an indicator of cerebrovascular health, and its signature in familial frontotemporal dementia (FTD) remains unknown. The primary aim was to investigate CVR in genetic FTD using an fMRI index of vascular contractility termed resting-state fluctuation amplitudes (RSFAs) and to assess whether RSFA differences are moderated by age. A secondary aim was to study the relationship between RSFA and cognition.

**Methods:**

Participants included presymptomatic and symptomatic *C9orf72*, *GRN*, and *MAPT* pathogenic variation carriers, along with noncarriers, from the prospective Genetic FTD Initiative cohort study. Cross-sectional differences in CVR were assessed using both component-based and voxel-level RSFA maps. To study disease progression–related effects, the moderating effect of age on differences between genetic status groups was analyzed using generalized linear models. The influence of RSFA, and its interaction with genetic status, on participants' cognitive function was also examined. All models were adjusted for sex, handedness, and scanning site and false discovery rate–corrected at *p* < 0.05.

**Results:**

A total of 284 presymptomatic and 124 symptomatic sequence variation carriers, and 265 noncarriers, were included in the analysis (mean age 48.17 years, 55% female). Across the sample, symptomatic carriers exhibited lower RSFA and a greater age-related RSFA decline predominantly in the medial frontal (−0.07 standard units, *p* = 0.046, 95% CI −0.13 to −0.01) and posterior parietal (−0.06 standard units, *p* = 0.048, 95% CI −0.12 to 0.01) cortex, compared with presymptomatic carriers and noncarriers. RSFA was inversely correlated with age (−0.43 standard units, *p* < 0.001, 95% CI −0.48 to −0.37) and positively associated with cognitive function (0.09 standard units, *p* = 0.008, 95% CI 0.04–0.15), particularly in the prefrontal cortex, in sequence variation carriers across the sample, independent of disease stage.

**Discussion:**

CVR impairment in genetic FTD has a predilection for the middle frontal and posterior cortex, and its preservation may yield a cognitive benefit for at-risk individuals. Although findings do not provide causality and warrant replication, they support the notion that vascular dysfunction in familial FTD may be a target for biomarker identification and disease-modifying efforts.

## Introduction

Frontotemporal dementia (FTD) encompasses heterogeneous neurodegenerative diseases. Multiple mutations in known Mendelian FTD genes are described, but most of the heritability is accounted for by autosomal dominant pathogenic variation in the genes chromosome 9 open reading frame 72 (*C9orf72*), progranulin (*GRN*), and microtubule-associated protein tau (*MAPT*).^1^ Prodromal FTD presents with neuropathologic changes decades before symptoms, including brain atrophy, disrupted white matter (WM) integrity, and functional connectivity, predominantly in fronto-temporo-parietal regions.^1-4^

Alongside tau and TDP-43–associated molecular pathologies, FTD involves cerebrovascular dysregulation. This includes impairments in the brain's neurovascular unit and blood-brain barrier (BBB), with damaged endothelial cells, dysfunctional pericytes,^5^ and associated secondary inflammation.^6^ Reduced cerebral blood flow (CBF), especially in the frontal cortex, is found in genetic FTD and correlates with impaired performance on neuropsychological tests.^3,4^ These findings, alongside small vessel pathology in autopsy-confirmed frontotemporal lobar degeneration (FTLD),^7^ imply comparable interaction between neurodegeneration and cerebrovascular impairment in FTD.

An important indicator of cerebrovascular function is cerebrovascular reactivity (CVR). CVR denotes the dilatory capacity of cerebral blood vessels in response to physiologic modulators, such as carbon dioxide, and regulates regional blood flow through pH-dependent vascular smooth muscle tone modulation.^8^ CVR is compromised by aging, vascular risk factors,^9^ and neurodegenerative conditions, such as Alzheimer disease (AD),^10,11^ suggesting that similar alterations may occur in FTD.

Traditional CVR mapping methods using hypercapnic agents or breath holding, although effective, are cumbersome, which limits their clinical applicability.^12,13^ This study adopts blood oxygenation–level dependent (BOLD) fMRI approach leveraging natural cardiorespiratory variations to extract a surrogate for arterial carbon dioxide fluctuations from resting-state data.^14-17^ Although resting-state BOLD data contain a variety of physiologic origins,^17^ previous efforts have studied acquisition and analysis schemes for reliable voxel-wise CVR estimation.^14,15^ Correlations of resting-state fMRI derivatives with traditional CVR mapping methods are moderate-to-high, ranging from *r* values of 0.36 at 3T^14,16,18,19^ to 0.96 at 7T MRI.^20^ Among resting-state techniques,^13-17^ resting-state fluctuation amplitudes (RSFA) offers robust within-participant reliability,^19^ cross-cohort reproducibility,^21-23^ and analytical consistency,^22,24^ without the need for invasive procedures or physiologic recordings.^17^ Previous studies suggest that group and individual RSFA differences do not reflect variations in neuronal activity, for example, from electro- or magneto-encephalography (M/EEG).^25^ Instead, these effects can be fully explained by a combination of cardiovascular and neurovascular signals.^22^ RSFA is non-invasive and can be extracted retrospectively from existing resting-state fMRI measures, suitable for large-scale studies with frail populations.^21,26^ It has been used to examine cardiovascular and cerebrovascular function in various conditions, including aging,^22,25^ AD,^27^ small vessel disease,^28^ Moyamoya disease,^14^ and hemodynamic impairment^23^ (an overview is provided in reference 26).

The principal aim was to determine the RSFA signature of presymptomatic and symptomatic genetic FTD. In addition, we assessed RSFA correlations with age and clinical status. We predicted reductions in RSFA in pathogenic variation carriers compared with pathogenic variation–negative family members and that these differences would increase with disease progression and relate to impaired cognitive performance.

## Methods

### Participants

Data were obtained from the prospective multicenter Genetic Frontotemporal Dementia Initiative (GENFI) cohort study. The sample included 680 individuals voluntarily recruited between January 2012 and May 2019 across 31 European and Canadian sites from families with a confirmed sequence variation in *C9orf72*, *GRN*, or *MAPT* genes. Individuals were either (1) symptomatic sequence variation carriers, (2) sequence variation carriers who did not exhibit any symptoms (i.e., presymptomatic), or (3) sequence variation–negative family members who served as controls, termed noncarriers. All participants were genotyped at their local site; a pathogenic expansion in *C9orf72* was defined as presence of greater than 30 repeats. Sequence carriers (affected and unaffected) were included if they completed at least 1 neuropsychological assessment. Individuals were considered symptomatic if their clinician considered evidence of progressive degenerative symptoms. The datasets of 7 participants were excluded because of motion-related or other imaging artifacts (3 symptomatic *C9orf72* carriers; 3 presymptomatic *GRN* carriers, and 1 mutation-negative individual from a GRN carrier family), resulting in a final sample of 673 participants.

### Neurocognitive Assessment and Indices of Cognitive Function

All participants underwent clinical evaluation, including medical and family history, functional status, and physical examination, corroborated by a close contact. They also completed a neuropsychological battery from the Uniform Data Set,^29^ assessing executive function (Digit Span Forward and Backward from the Wechsler Memory Scale–Revised; Parts A and B of the Trail Making Test; a Digit Symbol Task) and language (short version of the Boston Naming Test; Category Fluency [animals and combined]), and the Wechsler Abbreviated Scale of Intelligence Block Design Task. More details on the recruitment procedure and clinical assessment protocol are provided in another study.^30^

As a proxy for cognitive function, we performed principal component analysis (PCA) to derive a composite summary score across these cognitive assessments. The PCA technique helps minimize multiple comparison issues and reduces the dimensionality of cognitive function into 1 latent variable, with the largest proportion of shared variance as the first principal component (PC 1). Missing values were imputed using multivariate Markov Chain Monte Carlo imputation with chained equations with default settings in R.^31^

### Image Acquisition and Preprocessing

Structural MRI scans were obtained across 25 sites using a T1-weighted magnetization-prepared rapid gradient-echo sequence optimized for different manufacturers^30^ with acquisition parameters as follows: 1-mm median isotropic resolution; repetition time (TR) 2,000 milliseconds (2,000–2,200 milliseconds); echo time (TE) 2.9 milliseconds (2.8–4.6 milliseconds); inversion time 900 milliseconds (850–933 milliseconds); field of view 256 × 256 × 208 mm; minimum scanning time 283 seconds (283–462 seconds).

The T1-weighted images were analyzed using FSL and Statistical Parametric Mapping pipelines,^32,33^ including native-space segmentation of gray matter (GM), WM, and CSF tissue classes and voxel-wise morphometric analysis with Computational Anatomy Toolbox (CAT12)^34,35^ in Statistical Parametric Mapping (SPM12). Segmented images were modulated by Jacobian determinants with a DARTEL algorithm, normalized to the Montreal Neurological Institute (MNI) template, and analyzed voxel-wise with the Commonality toolbox for neuroimaging.^36^ More information about structural MRI data processing is provided in eMethods 1.

Resting-state fMRI data were acquired using echo-planar imaging (EPI) sequences harmonized across GENFI sites.^30^ Parameters included the following: TR 2,500 milliseconds (2,200–2,500 milliseconds), TE 30 milliseconds, flip angle 80° (80°–85°), in-plane resolution 2.72 × 2.72 mm, and 3.5-mm slice thickness. Participants were instructed to lay still with eyes closed. The first 6 volumes were discarded for T1 equilibration. Motion was quantified through root mean square volume-to-volume displacement.^37^ The preprocessing, performed using SPM12 in MATLAB R2021b (MathWorks, Natick, MA),^38^ comprised spatial realignment, slice-time correction to the middle slice, co-registration of EPI to T1 scans, normalization to MNI space, and smoothing with an 8-mm Gaussian full-width at half-maximum kernel. Resting-state time series were further processed using data-driven independent component analysis (ICA)^39^ to reduce noise confounding,^40^ detrending of the fMRI signal, regression of motion, WM and CSF signals, their derivative and quadratic regressors,^41^ and band-pass filtering (0.0078–0.01 Hz). Signals from WM and CSF were estimated using the average of WM and CSF masks derived by thresholding SPM's corresponding tissue probability maps at 0.75. RSFA was defined as the voxel-wise normalized standard deviation across time of these processed time series. Details on the EPI data processing are available in eMethods 2.

### Indices of Cerebrovascular Function Using RSFA

To evaluate RSFA differences between groups and disease progression, we used multivariate and univariate approaches. We used ICA to identify spatially independent CVR patterns without a priori hypotheses. ICA offers advantages over univariate methods by mitigating multiple comparison issues, while capturing both widespread and localized latent data features that often characterize complex neurologic conditions.^24,42^ We complemented ICA by voxel-wise analysis to detect localized RSFA differences with high spatial resolution.

### Component-Based Analysis

Spatial ICA was implemented using the Source-Based Morphometry toolbox^42^ in the Group ICA for fMRI Toolbox.^43^ The optimal number of sources was identified by PCA with minimum description length (MDL) criterion.^44^ The data were decomposed into spatially independent components (i.e., “IC maps”) with associated standardized participant-specific scores. Components' reliability was confirmed using the ICASSO tool.^45^ Components with high reliability confined to GM areas, considered indicative of vascular reactivity^22^ and linked to cognitive function,^46^ were regarded as relevant for subsequent analyses. Full details on the ICA implementation are described in eMethods 3.

### Voxel-Based Univariate Analysis

For completeness, we also conducted voxel-wise analysis of RSFA maps using a voxel-based general linear model–like approach implemented in the Commonality Analysis library in MATLAB.^47^ This method enables analysis of localized RSFA differences while controlling for voxel-specific covariates, such as GM volume. Statistically significant clusters where between-group effects were observed were used to define regions of interest (ROIs) and visualize group differences.

### Statistical Analysis

#### Descriptive Statistics

Demographic characteristics were compared with IBM SPSS Statistics for Windows (version 29.0; IBM Corp., Armonk, NY, released 2021). Welch analysis of variance with Games-Howell post hoc tests was used for continuous data and the χ^2^ test for categorical variables. The significance level was defined as 2-tailed with a threshold at *p* = 0.05.

#### FTD-Related Effects on Cerebrovascular Indices Using RSFA

Cross-sectional RSFA differences between symptomatic and presymptomatic carriers (all sequence variations combined) and noncarriers were examined on component-based estimates of RSFA using robust multiple linear regression (MLR) (MATLAB function *fitlm.m*). In these models, IC subject scores for each component (termed RSFA_ICn_, where *n* denotes the number of the selected component) were the dependent variable, with age, sex, and handedness as covariates of no interest. Scanning site was included as a covariate of no interest to adjust across scanning platforms. The study's analytical approach is presented in eFigure 1.

To explore disease progression effects across genetic status groups, we also investigated the moderating effect of age on the case-control differences. Model formulas were specified by Wilkinson notation, for example, Model 1: “RSFA_IC_ ∼1 + genetic status × age + sex + handedness + scanning site,” providing a flexible way to examine main effects of predictors of interest (genetic status and age) and their interaction (genetic status × age), while adjusting for confounders of no interest. To account for multiple testing issues, the overall model fit was corrected using the Benjamini-Hochberg false discovery rate (FDR) procedure at 0.05 level.

Finally, to control for potential contribution of brain atrophy to the RSFA effects, an average of regional GM volume was computed for each RSFA IC map. The regional GM values per component were entered as covariates within the same statistical model (model 2: “RSFA_IC_ ∼1 + genetic status × age + GM_IC_ + sex + handedness + scanning site”).

We further explored the distribution of RSFA effects using voxel-wise analysis within the same model (e.g., Model 1: “RSFA_Voxel_ ∼1 + genetic status × age + sex + handedness + scanning site”). We used nonparametric testing as part of the voxel-based Commonality Analysis library in MATLAB, which facilitates univariate neuroimaging analysis.^47^ Significant clusters were identified with nonparametric testing using 5,000 permutations and threshold-free cluster enhancement (TFCE) with 0.01 significance level,^48^ unless otherwise specified. The pipeline is available online.^49^

Clusters exhibiting between-group differences after TFCE correction were also adjusted for GM volume per cluster, considering potential confounding effects of atrophy on RSFA. To correct for multiple comparisons, we controlled the voxel-level FDR at *p* < 0.05. Significant clusters at TFCE level were used to define ROIs for exploring associations between RSFA and age by genetic status. Post hoc tests compared noncarriers vs symptomatic carriers, noncarriers vs presymptomatic carriers, and presymptomatic carriers vs symptomatic carriers. Regions were labeled according to the Automated Anatomical Labeling Atlas.^50^

#### Behavioral Relevance of Cerebrovascular Impairment

A secondary objective of this study was to evaluate the behavioral relevance of RSFA to cognitive function. Differences in cognitive performance scores between genetic status groups were explored using the Kruskal-Wallis test with Mann-Whitney post hoc tests. Subsequent regression models included global cognitive function, represented by participant scores for PC 1 from the PCA, as the dependent variable. Independent variables included the RSFA_IC_ for each neurocognitively meaningful component and RSFA in representative ROIs from TFCE-corrected voxel-wise analysis. Interaction terms assessed whether the RSFA-cognition association varied by genetic status, while adjusting for age, sex, handedness, and scanning site. For completeness, regression models were re-ran using domain-specific cognitive scores that loaded most strongly on PC 1. These included the Trail Making Test Parts A and B, Digit Symbol Task, and Verbal Fluency, suggesting that PC 1 represented most prominently executive function. Details about each principal component are presented in eFigures 2 and 3 and eTable 1.

Model formulas took the following form: Model 3: “Cognition_PC1_ ∼ 1 + genetic status × RSFA_IC/Voxel_ + age + sex + handedness + scanning site.” FDR correction was applied (FDR <0.05), and post hoc tests between subgroups of interest were performed for any established main effects (eFigure 1).

### Standard Protocol Approvals, Registrations, and Patient Consents

Informed consent was obtained from all human participants. The study was given a favorable opinion by the Cambridge 2 Research Ethics Committee REC 17/EE/0032 IRAS ID 204052.

### Data Availability

Data were acquired from GENFI data freeze 5. Anonymized data not published within this article will be made available by request from any qualified investigator and can be requested through the GENFI website (genfi.org/contact-us-2) or through Dementias Platform UK (portal.dementiasplatform.uk/Apply).

## Results

### Demographics

Characteristics of the sample are presented in Table 1. A total of 673 participants were included in the study—124 symptomatic (61 *C9orf72*, 40 *GRN*, and 23 *MAPT*) sequence variation carriers, 284 presymptomatic (107 *C9orf72*, 123 *GRN*, and 54 *MAPT*) carriers, and 265 noncarriers. The mean age (standard deviation, SD) of sequence variation–negative family members was 48.17 (13.43) years and of presymptomatic sequence variation carriers was 45.95 (13.09) years, compared with symptomatic carriers whose mean age was 62.64 (7.43) years. There were more females than males among noncarriers (153–112) and presymptomatic carriers (165–119) compared with symptomatic individuals (53–71). No significant differences were observed between noncarriers and presymptomatic carriers for the remaining demographic variables.

**Table 1:** Demographic Information of Participants Included in the Analysis, Grouped by Genetic Status as Non-carriers, Pre-symptomatic Carriers, and Symptomatic Carriers

Demographics	Sample	NC	PSC	SC	Group comparison, *p* value^a^			
					Sample	NC vs SC	PSC vs SCC	NC vs PSC
Total, N	673	265 (39.38)	284 (42.2)	124 (18.42)				
Sequence variation in family					0.126			
*C9orf72*	264 (39.23)		107	61				
*GRN*	276 (41.01)		123	40				
*MAPT*	133 (19.76)		54	23				
Age, y	48.17 ± 13.43	45.95 ± 13.09	43.93 ± 11.4	62.64 ± 7.43	<0.001	<0.001	<0.001	0.132
Sex ratio (F:M)	371:302	153:112	165:119	53:71	0.009	0.006	0.004	0.931
Estimated years from onset	−10.62 ± 13.40	−13.21 ± 13.47	−14.30 ± 11.63	3.32 ± 6.24	<0.001	<0.001	<0.001	0.569
Education, y	14.18 ± 3.45	14.51 ± 3.35	14.50 ± 3.36	12.72 ± 3.53	<0.001	<0.001	<0.001	0.998

Abbreviations: *C9orf72* = chromosome 9 open reading frame 72; *GRN* = progranulin; *MAPT* = microtubule-associated protein tau; NC = noncarrier; PSC = presymptomatic carrier; SC = symptomatic carrier of a sequence variation.

Values indicate count (%) or mean ± SD.

a *p* Values are the result of the *F* test or χ^2^ test, as appropriate. Statistical significance was at *p* < 0.05. Years to expected onset is defined as the difference between age at assessment and mean age at onset within the family and is provided for descriptive purposes.

### Regional Differences in RSFA Based on Independent Component Analysis

**Figure 1:**
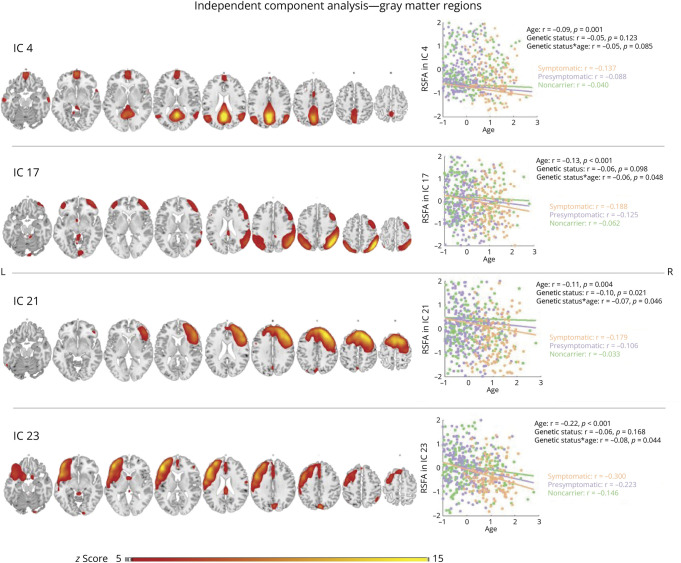
Neurocognitively Meaningful Independent Components Based on Spatial ICA on RSFA Maps Spatial distribution of 4 ICs within neurocognitively meaningful areas (i.e., GM regions) based on spatial ICA on RSFA maps across participants, where differences in IC loading values are found in association with genetic status, age, and genetic status × age interaction. Robust general linear model regression lines for each IC are presented in scatter plots with respective *r* values on the right side of each IC map. *p* Values are FDR-corrected at the 0.05 level across the whole sample. Group-level spatial maps are overlaid onto the Colin-27 (ch2.nii) structural template of the MNI brain, where intensity values correspond to z-values. FDR = false discovery rate; GM = gray matter; IC = independent component; ICA = IC analysis; MNI = Montreal Neurological Institute; RSFA = resting-state fluctuation amplitudes.

Applying ICA with MDL criterion to the RSFA data yielded 24 components, indicating signal from GM regions, CSF, vasculature, and other nonphysiologic factors (eFigure 4). Twenty components were excluded (eTable 2). The overall model fit of 4 GM components remained significant after FDR correction (Figure 1). Key voxels included posterior cingulate cortex (PCC)/precuneus (IC 4), posterior association and parieto-occipital association areas (IC 17), and right (IC 21) and left (IC 23) lateral prefrontal cortex (PFC) (Figure 1). A tendency of FTD-dependent decrease in RSFA was found for all components across the sample. Significant RSFA reduction was revealed in component 21, driven by differences between symptomatic carriers and noncarriers, and presymptomatic and symptomatic carriers. In addition, significant genetic status × age interaction was demonstrated across the sample for components IC 17, IC 21, and IC 23, with symptomatic carriers displaying steeper age-related RSFA decreases, followed by presymptomatic carriers and sequence variation–negative individuals. This suggests greater age-related RSFA decline in at-risk and affected participants, potentially exacerbating disease progression. Figure 1 presents spatial maps with IC participant scores, and Table 2 provides these results. Insertion of GM as a covariate of no interest into the models did not alter findings substantially, highlighting the specificity of RSFA effects (eTable 3).

**Table 2:** Multiple Regression Results of IC Subject Loadings From Independent Component Analysis Across Groups of Interest

Model 1: “RSFA_IC_ ∼ 1 + genetic status × age + sex + handedness + scanning site”										
Predictor	Adjusted *R*^2^	Age			Genetic status			Genetic status × age		
		β (95% CI)	*t*	*p* Value^a^	β (95% CI)	*t*	*p* Value^a^	β (95% CI)	*t*	*p* Value^a^
IC 4: posterior cingulate cortex/precuneus										
Sample	0.62	−0.09 (−0.14 to −0.04)	−3.59	0.001	−0.05 (−0.10 to 0.01)	−1.77	0.123	−0.05 (−0.11 to 0)	−2.00	0.085
SC vs NC		−0.08 (−0.17 to 0.01)	−1.83	0.069						
PSC vs SC		−0.13 (−0.21 to −0.05)	−3.18	0.002						
PSC vs NC		−0.07 (−0.12 to −0.02)	−2.63	0.009						
IC 17: posterior parietal association areas										
Sample	0.54	−0.13 (−0.18 to −0.07)	−4.27	<0.001	−0.06 (−0.12 to 0.01)	−1.80	0.098	−0.06 (−0.12 to 0.01)	−2.17	0.048
SC vs NC		−0.16 (−0.26 to −0.06)	−3.08	0.002				−0.14 (−0.26 to −0.01)	−2.18	0.030
PSC vs SC		−0.22 (−0.31 to −0.12)	−4.37	<0.001				−0.10 (−0.22 to 0.01)	−1.76	0.079
PSC vs NC		−0.09 (−0.15 to −0.03)	−3.07	0.002				−0.03 (−0.09 to 0.03)	−1.13	0.258
IC 21: right lateral prefrontal cortex										
Sample	0.44	−0.11 (−0.17 to −0.04)	−3.33	0.004	−0.10 (−0.17 to 0.03)	−2.68	0.021	−0.07 (−0.13 to −0.01)	−2.31	0.046
SC vs NC		−0.04 (−0.14 to 0.01)	−0.70	0.487	−0.24 (−0.36 to 0.11)	−3.67	<0.001	0.01 (−0.12 to 0.14)	0.11	0.915
PSC vs SC		−0.06 (−0.17 to 0.04)	−1.16	0.247	−0.22 (−0.35 to 0.08)	−3.13	0.002	0.02 (−0.10 to 0.15)	0.35	0.726
PSC vs NC		−0.06 (−0.12 to 0.01)	−1.69	0.092	−0.01 (−0.08 to 0.05)	−0.33	0.742	−0.01 (−0.08 to 0.05)	−0.42	0.677
IC 23: left lateral prefrontal cortex										
Sample	0.49	−0.22 (−0.28 to −0.16)	−7.22	<0.001	−0.06 (−0.13 to 0)	−1.87	0.168	−0.08 (−0.14 to −0.02)	−2.52	0.044
SC vs NC		−0.13 (−0.23 to −0.02)	−2.42	0.016				−0.01 (−0.14 to 0.11)	−0.19	0.846
PSC vs SC		−0.20 (−0.30 to −0.10)	−4.08	<0.001				0.07 (−0.04 to 0.19)	1.23	0.220
PSC vs NC		−0.18 (−0.25 to −0.12)	−5.41	<0.001				−0.05 (−0.12 to 0.01)	−1.61	0.108

Abbreviations: FDR = false discovery rate; GM = gray matter; IC = independent component; NC = noncarrier; PSC = presymptomatic carrier; RSFA = resting-state fluctuation amplitudes; SC = symptomatic carrier of a sequence variation.

RSFA differences are shown across groups of interest after robust multiple linear regression analysis on component-based RSFA maps. Estimated regression parameters, *t* values, and *p* values are shown for main effects across the entire sample and post hoc tests between subgroups of interest where relevant. β (95% CI) denote standardized (β) coefficients with 95% lower and upper CIs. Outcomes of interest are the RSFA-IC loadings associated with ICA components within GM regions where case-control differences are found. Models are adjusted for sex, handedness, and scanning site.

a *p* Values are FDR-corrected at the 0.05 level in comparisons across the whole sample (all genetic status groups combined).

Last, ICA also revealed components originating from large blood vessels, venous drainage sites, and CSF (eFigure 4). They tended to display higher subject scores in older (symptomatic) individuals, likely reflecting vascular health differences and other physiologic factors.^26,28^

### Spatial Distribution and Voxel-Wise Univariate Differences in RSFA

**Figure 2:**
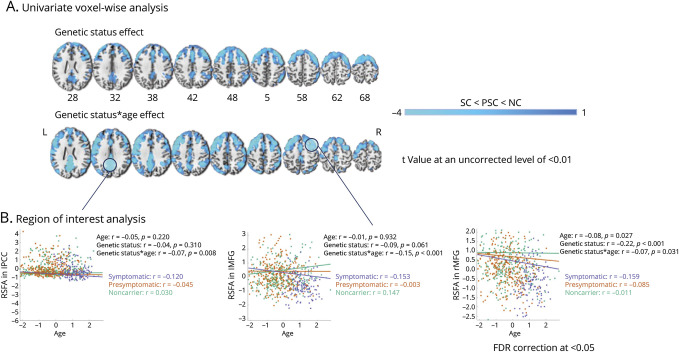
Differences in Global Cognitive Function in Association With Genetic Status, RSFA, and Genetic Status × RSFA Interaction Across Groups of Interest Cognitive function is denoted by participants' loading values for PC 1 after PCA on 9 cognitive measures. Effects are illustrated for ICA-based components within GM areas (A) and several representative ROIs based on TFCE-corrected voxel-wise univariate analysis (B). Robust general linear model regression lines for each respective IC and ROI are presented in scatter plots with corresponding *r* values on the right side of a representative slice depicting each IC/ROI map. *p* Values are FDR-corrected at the 0.05 level across the whole sample. FDR = false discovery rate; GM = gray matter; IC = independent component; ICA = IC analysis; MFG = middle frontal gyrus; PC = principal component; PCA = PC analysis; PCC = posterior cingulate cortex; ROI = region of interest; RSFA = resting-state fluctuation amplitudes; TFCE = threshold-free cluster enhancement.

Overall, voxel-based analysis results were consistent with those of component-based analysis, particularly in frontal cortical and posterior parietal regions. Group-level analysis across all genetic groups revealed age-related RSFA decreases in clusters including left precuneus, right cuneus, left inferior parietal lobule, left precentral gyrus, and right superior frontal gyrus (SFG). In voxel-wise analysis, genetic status–dependent RSFA reduction was observed in the bilateral middle frontal gyrus (MFG) and SFG, with symptomatic carriers exhibiting greater RSFA decline, compared with presymptomatic carriers and noncarriers. Lower RSFA was found as a function of age × genetic status interaction in left precuneus/PCC. Conversely, RSFA increases, related to age and genetic status, were also observed in clusters spanning cerebellum and subcortical regions, including thalamus and putamen. The anatomical localization of the clusters is presented in Table 3 and visualized in Figure 2. Complete information about all voxel-wise clusters is provided in eTable 4. Inclusion of regional GM into the models helped explain unique RSFA effects (eTable 5 and eFigure 5) but, importantly, did not alter the main results, consistent with ICA findings, which implies that regional atrophy effects do not explain the RSFA reductions.

**Table 3:** Anatomical Localization of Voxel-Wise Multiple Regression Analysis–Derived Clusters Significant at TFCE Level Where RSFA Differences Are Observed Across the Sample

Contrast name	Cluster name	Peak *t* score	Peak *p* value	MNI coordinates (mm)		
SC > PSC > NC						
Age	L. Precuneus	−8.97	0.0002	−4	−80	44
	L. Inferior parietal lobule	−7.51	0.0002	−58	−32	44
	R. Cuneus	−6.45	0.0002	12	−70	34
	L. Precentral gyrus	−6.27	0.0002	−50	12	34
	R. Superior frontal gyrus	−6.27	0.0002	2	32	56
Genetic status	R. Middle frontal gyrus	−5.91	0.002	40	26	46
	R. Superior frontal gyrus	−4.71	0.005	26	6	70
	L. Middle frontal gyrus	−4.71	0.009	−28	32	52
	L. Superior frontal gyrus	−4.38	0.009	−16	2	74
	R. Inferior frontal gyrus	−3.97	0.046	54	18	24
Genetic status × age	L. Posterior cingulate cortex	−5.14	0.011	−4	−44	24
	L. Precuneus	−4.36	0.023	−4	−66	26

Abbreviations: MNI = Montreal Neurological Institute; NC = noncarrier; PSC = presymptomatic carrier; RSFA = resting-state fluctuation amplitudes; SC = symptomatic carrier of a sequence variation; TFCE = threshold-free cluster enhancement.

RSFA differences are observed across the whole sample (all genetic status groups combined) after robust multiple linear regression analysis on RSFA maps in statistically significant clusters of interest at TFCE level. Each TFCE cluster is represented by its name according to the Anatomical Labeling Atlas and corresponding coordinates in MNI space. Significance was determined based on a null distribution of 5,000 permutations and TFCE with a significance level of 0.01. The method takes a raw statistic image and produces an output image in which voxel-wise values represent the amount of cluster-like local spatial support; that is, the output value is a weighted sum of the entire local clustered signal, without the need to arbitrarily define an initial cluster-forming threshold value. For inference, the TFCE image is turned into voxel-wise *p* values that can be corrected for multiple comparisons across space through permutation testing; hence, no estimates and CIs are presented for these results. Models are adjusted for sex, handedness, and scanning site.

**Figure 3:**
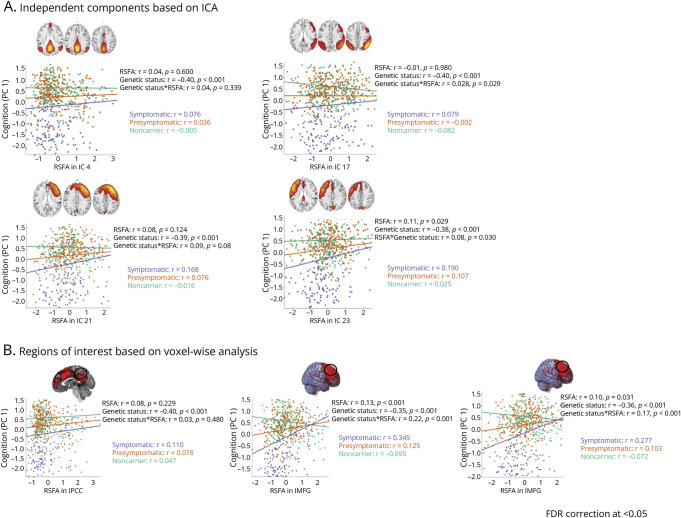
RSFA Effects Based on Voxel-Wise Univariate Analysis (A) Regional distribution of RSFA effects based on voxel-level univariate analysis. Cold colors denote RSFA decreases as a function of genetic status and their interaction with age. Statistical parametric maps are displayed at an uncorrected level of *p* < 0.01 to better visualize regional CVR patterns. Images are overlaid onto the Colin-27 (ch2.nii) structural template of the MNI brain. (B) Differences in RSFA in association with genetic status, age, and genetic status × age interaction across groups of interest in several representative ROIs based on TFCE-corrected voxel-wise univariate analysis on RSFA maps. Robust general linear model regression lines for each ROI are presented in scatter plots with respective *r* values on the right side of each ROI map. *p* Values are FDR-corrected at the 0.05 level across the whole sample. CVR = cerebrovascular reactivity; FDR = false discovery rate; MFG = middle frontal gyrus; MNI = Montreal Neurological Institute; NC = noncarrier; PCC = posterior cingulate cortex; PSC = presymptomatic carrier; ROI = region of interest; RSFA = resting-state fluctuation amplitudes; SC = symptomatic carrier of a sequence variation; TFCE = threshold-free cluster enhancement.

Regression analysis in several representative ROIs from TFCE-corrected voxel-wise clusters, including left precuneus/PCC and bilateral MFG, demonstrated lower RSFA as a function of age and genetic status group in sequence variation carriers compared with noncarriers (Figure 3). The largest differences existed between symptomatic carriers and noncarriers, and presymptomatic carriers and noncarriers (post hoc tests are summarized in eTable 6).

Lastly, we compared RSFA-IC loadings and RSFA-ROI estimates between groups stratified by mutated gene. No between-group differences were detected based on sequence variation(eTable 7).

### Relationship Between RSFA and Cognition

PCA showed that PC 1 explained 62% of the variance in cognitive performance; PC 2 and PC 3 accounted for 9% and 7%, respectively (eFigure 2 and eTable 1). We focused on PC 1 as a proxy for cognitive function. A Kruskal-Wallis test revealed significant differences in PC 1 scores between genetic status groups (χ^2^(2) = 256.02, *p* < 0.001). Post hoc Mann-Whitney tests indicated lower cognitive function (i.e., lower PC 1 scores), in symptomatic carriers compared with presymptomatic carriers (*U* = 1,461, *p* < 0.001) and noncarriers (*U* = 1,182, *p* < 0.001). No significant difference was present between presymptomatic carriers and noncarriers (*U* = 36,318, *p* = 0.480).

Regression analysis showed a positive relationship between RSFA in left PFC (IC 23) and global cognitive function (PC 1), indicating better overall cognitive performance in individuals with higher RSFA. In addition, a genetic status × RSFA interaction was observed in the same component, posterior parietal association areas (IC 17), and right lateral PFC (IC 21), with stronger association between RSFA and global cognition in sequence variation carriers, particularly symptomatic participants, than in noncarriers (Figure 2). ROI analysis was consistent with component-based analysis. The output from the MLR models is presented in Table 4. Results with domain-specific cognitive scores aligned with PCA findings (eTable 8). This underscored the role of RSFA in the frontal cortex in maintaining cognitive function in individuals at genetic risk of FTD.

**Table 4:** Multiple Regression Results of Global Cognition as a Function of RSFA

Model 3: “Cognition _PC1_ ∼ 1 + genetic status × RSFA_IC/Voxel_ + age + sex + handedness + scanning site”												
	Sample			SC vs NC			PSC vs SC			PSC vs NC		
	β (95% CI)	*t*	*p* Value^a^	β (95% CI)	*t*	*p* Value^a^	β (95% CI)	*t*	*p* Value^a^	β (95% CI)	*t*	*p* Value^a^
ICs based on ICA “Cognition_PC1_ ∼ 1 + genetic status × RSFA_IC_ + age + sex + handedness + scanning site”												
IC 4: posterior cingulate cortex/precuneus; model-adjusted *R*^2^ = 0.52												
Age	−0.43 (−0.49 to −0.37)	−14.10	<0.001	−0.23 (−0.30 to −0.16)	−6.36	<0.001	−0.30 (−0.38 to −0.23)	−7.69	<0.001	−0.37 (−0.45 to −0.30)	−9.45	<0.001
Genetic status	−0.40 (−0.47 to −0.34)	−13.07	<0.001	−0.68 (−0.75 to −0.60)	−18.09	<0.001	−0.61 (−0.68 to −0.53)	−15.28	<0.001	−0.01 (−0.08 to 0.07)	−0.19	0.851
RSFA	0.04 (−0.05 to 0.12)	0.80	0.600									
Genetic status × RSFA	0.04 (−0.02 to 0.10)	1.43	0.339									
IC 17: posterior association areas; model-adjusted *R*^2^ = 0.52												
Age	−0.44 (−0.50 to −0.38)	−14.23	<0.001	−0.23 (−0.30 to −0.16)	−6.46	<0.001	−0.30 (−0.38 to −0.23)	−7.67	<0.001	−0.37 (−0.45 to −0.29)	−9.38	<0.001
Genetic status	−0.40 (−0.46 to −0.34)	−13.05	<0.001	−0.68 (−0.76 to −0.61)	18.21	<0.001	−0.61 (−0.69 to −0.53)	−15.28	<0.001	−0.01 (−0.09 to 0.06)	−0.29	0.769
RSFA	−0.01 (−0.08 to 0.08)	−0.04	0.980									
Genetic status × RSFA	0.08 (0.02 to 0.14)	2.84	0.029	0.04	1.33	0.183	0.02 (−0.04 to 0.08)	0.53	0.600	0.07 (−0.01 to 0.14)	1.77	0.078
IC 21: right lateral prefrontal cortex; model-adjusted *R*^2^ = 0.53												
Age	−0.43 (−0.48 to −0.37)	−14.08	<0.001	−0.23 (−0.30 to −0.16)	−6.55	<0.001	−0.30 (−0.37 to −0.22)	−7.76	<0.001	−0.37 (−0.45 to −0.29)	−9.40	<0.001
Genetic status	−0.39 (−0.45 to −0.33)	−12.61	<0.001	−0.67 (−0.74 to −0.60)	−17.74	<0.001	−0.60 (−0.68 to −0.52)	−14.73	<0.001	−0.01 (−0.08 to 0.07)	−0.16	0.869
RSFA	0.08 (0 to 0.15)	2.06	0.124									
Genetic status × RSFA	0.09 (0.04 to 0.15)	3.40	0.008	0.05 (−0.01 to 0.11)	1.91	0.057	0.02 (−0.04 to 0.08)	0.64	0.523	0.05 (−0.03 to 0.12)	1.26	0.207
IC 23: left lateral prefrontal cortex; model-adjusted *R*^2^ = 0.52												
Age	−0.40 (−0.46 to −0.34)	−12.85	<0.001	−0.22 (−0.29 to −0.15)	−6.05	<0.001	−0.29 (−0.36 to −0.21)	−7.24	<0.001	−0.36 (−0.44 to −0.28)	−8.92	<0.001
Genetic status	−0.38 (−0.45 to −0.32)	−12.33	<0.001	−0.67 (−0.75 to −0.60)	−17.51	<0.001	−0.59 (−0.67 to −0.51)	−14.28	<0.001	−0.01 (−0.08 to 0.07)	−0.19	0.846
RSFA	0.11 (0.03 to 0.18)	2.88	0.029	0.07 (−0.01 to 0.15)	1.69	0.091	0.07 (−0.01 to 0.16)	1.76	0.079	0.07 (−0.03 to 0.17)	1.38	0.169
Genetic status × RSFA	0.08 (0.03 to 0.14)	2.83	0.030	0.01 (−0.05 to 0.07)	0.21	0.831	0.03 (−0.03 to 0.10)	1.06	0.289	−0.04 (−0.11 to 0.04)	−0.90	0.367
ROIs based on voxel-wise analysis “Cognition_PC1_ ∼ 1 + genetic status × RSFA_Voxel_ + age + sex + handedness + scanning site”												
Left posterior cingulate cortex/precuneus; model-adjusted *R*^2^ = 0.52												
Age	−0.43 (−0.49 to −0.37)	−14.14	<0.001	−0.23 (−0.30 to −0.16)	−6.42	<0.001	−0.30 (−0.38 to −0.22)	−7.66	<0.001	−0.38 (−0.46 to −0.30)	−9.45	<0.001
Genetic status	−0.40 (−0.46 to −0.34)	−12.91	<0.001	−0.67 (−0.75 to −0.60)	−17.87	<0.001	0.61 (−0.68 to −0.53)	−15.28	<0.001	−0.01 (−0.08 to 0.07)	−0.16	0.873
RSFA	0.08 (−0.01 to 0.17)	1.77	0.229									
Genetic status × RSFA	0.03 (−0.03 to 0.09)	1.05	0.480									
Left middle frontal gyrus; model-adjusted *R*^2^ = 0.57												
Age	−0.38 (−0.44 to −0.32)	−12.95	<0.001	−0.21 (−0.27 to −0.14)	−6.03	<0.001	−0.29 (−0.36 to −0.21)	−7.66	<0.001	−0.36 (−0.44 to −0.28)	−9.18	<0.001
Genetic status	−0.35 (−0.41 to −0.29)	−11.50	<0.001	−0.60 (−0.68 to −0.53)	−15.20	<0.001	−0.52 (−0.61 to −0.44)	−12.46	<0.001	−0.01 (−0.08 to 0.07)	−0.04	0.964
RSFA	0.13 (0.07 to 0.18)	4.31	<0.001	0.09 (0.02 to 0.15)	2.60	0.010	0.14 (0.08 to 0.20)	4.26	<0.001	−0.02 (−0.10 to 0.06)	−0.55	0.582
Genetic status × RSFA	0.22 (0.16 to 0.28)	7.60	<0.001	0.20 (0.13 to 0.27)	5.97	<0.001	0.15 (0.08 to 0.22)	4.41	<0.001	0.07 (0 to 0.15)	1.86	0.064
Right middle frontal gyrus; model-adjusted *R*^2^ = 0.55												
Age	−0.40 (−0.46 to −0.35)	−13.51	<0.001	−0.24 (−0.31 to −0.17)	−6.68	<0.001	−0.30 (−0.37 to −0.22)	−7.67	<0.001	−0.37 (−0.44 to −0.29)	−9.25	<0.001
Genetic status	−0.36 (−0.42 to −0.29)	−11.10	<0.001	−0.64 (−0.72 to −0.55)	−15.00	<0.001	−0.57 (−0.65 to −0.49)	−13.32	<0.001	−0.01 (−0.08 to 0.07)	−0.15	0.883
RSFA	0.10 (0.03 to 0.17)	2.82	0.031	0.04 (−0.04 to 0.11)	0.88	0.377	0.09 (0.01 to 0.17)	2.30	0.022	−0.02 (−0.12 to 0.08)	−0.34	0.737
Genetic status × RSFA	0.17 (0.12 to 0.23)	6.47	<0.001	0.10 (0.03 to 0.16)	3.02	0.003	0.04 (−0.02 to 0.10)	1.38	0.167	0.07 (−0.01 to 0.15)	1.83	0.067

Abbreviations: FDR = false discovery rate; ICA = independent component analysis; NC = noncarrier; PC = principal component; PCA = PC analysis; PSC = presymptomatic carrier; ROI = region of interest; RSFA = resting-state fluctuation amplitudes; SC = symptomatic carrier of a sequence variation

Cognitive function differences are observed as a function of RSFA and genetic status after robust multiple linear regression analysis in ICA-based components (top panel) and several representative ROIs derived from significant clusters in TFCE-corrected voxel-based univariate analysis on RSFA maps (bottom panel). Estimated regression parameters, *t* values, and *p* values are shown for main effects across the entire sample and subgroups of interest where relevant. β (95% CI) denote standardized (β) coefficients with 95% lower and upper CIs. Models are adjusted for age, sex, handedness, and scanning site. The outcome of interest is cognitive function, represented by participants' loading values for PC 1 after PCA on 9 cognitive measures (global cognition).

a *p* Values are FDR-corrected at the 0.05 level in comparisons across the whole sample (all genetic status groups combined).

Of note, most participants underwent cognitive assessment on the same day as their resting fMRI scan, although some discrepancies occurred (eTable 9 and eFigure 6). We adjusted models for differences between RSFA acquisition and cognitive evaluation, and the RSFA-cognition effects remained unchanged (eTable 10). We also explored RSFA-cognition group differences based on sequence variation but uncovered no significant effects at FDR-corrected levels (eTable 11 and eFigure 7).

## Discussion

We discovered reduced CVR, quantified using RSFA, by sequence variation associated with familial FTD even in the long presymptomatic period. The RSFA differences worsened with disease progression and correlated with cognition in affected carriers, beyond the effects of aging. We propose that cerebrovascular function is a dysregulated feature in the pathophysiology of FTD, including its prodrome, which may interact with neurodegenerative changes.

Progressive reductions in RSFA were exhibited in carriers of sequence variation vs noncarriers in the ventromedial and lateral PFC, cingulate cortex, and parietal cortex. Comparable RSFA decreases are reported in healthy aging and microvascular impairment, particularly in prefrontal, cingulate, and superior-parietal cortical areas^22,23,25^ that are vulnerable to atrophy^1,2,30^ and hypoperfusion^3,4^ in familial FTD. These areas have also shown abnormal vasoreactivity in AD^10,11^ and constitute parts of the default mode and salience networks, implicated in executive function and cognitive-affective regulation, each functionally impaired in genetic FTD.^1,30^

We argue that these RSFA decreases indicate cerebrovascular dysfunction that cannot be explained by neuronal loss, given that regional GM inclusion into the analyses did not alter the age-dependent and genetic status–dependent RSFA effects. Potential causes for reduced CVR include pH dysregulation and impaired nitric oxide modulation, which may diminish endothelium-dependent dilator responses and the dynamic range of the BOLD signal.^7,8^ Studies in FTLD and familial FTD have documented neurovascular alterations, including dysfunctional endothelium,^7^ depleted pericytes,^5^ and activated microglia.^6^ Given the interrelatedness between neurons and cerebral microvessels, such changes likely dysregulate the BBB, diminish brain perfusion,^3,4^ and trigger aberrant protein aggregation and neuroinflammation, accelerating neurodegeneration. Alternatively, the CVR changes may be independent of early neurodegeneration, suggesting that cerebrovascular dysfunction could be an interacting contributor to FTD etiology. This might explain the lack of atrophy effects on RSFA if cerebrovascular impairment occurs in areas where atrophy is not sufficiently advanced. By contrast, the RSFA increases in cerebellar and subcortical regions possibly reflect increased pulsatility in neighboring vascular and WM territories.^22,23,26,28^ Overall, these findings underscore the need to further discern the link between cerebrovascular alterations and neurodegenerative processes in FTLD pathologies.

The RSFA variances in middle frontal and posterior parietal/cingulate areas, consistent across ICA (ICs 4 and 17) and voxel-based analyses, accord with FTD-related hypoperfusion and atrophy profiles.^2-4,30^ However, the notable RSFA reductions in the lateral PFC (ICs 21 and 23) are not common in early FTD. This discrepancy implies that a shared pathway may impair CVR in the inferior and middle frontal and parietal areas affected by hypoperfusion and atrophy, alongside independent vascular deficits in dorsolateral frontal areas. RSFA effects in some ICA-identified regions may reflect multiple sources with different etiologies, highlighting the challenge of using univariate methods to dissociate spatially overlapping signal sources and supporting data-driven, multimodal approaches.^24,42^

Although we observed diminished RSFA in signature FTD frontal and parietal areas, no substantial RSFA decreases were discovered in temporal regions, despite their prominent involvement, especially in *MAPT* sequence variation.^1,2,30^ The RSFA comparisons across sequence variants did not reveal significant between-group differences. Potentially, this reflects small and unbalanced subgroups per gene variant or shared vascular co-pathology downstream of the genetic variants' molecular signatures. The frontal RSFA reductions may be due to distinct mechanisms from atrophy and perfusion alterations previously uncovered in FTD.^2-4,30^ In line with this assumption, forebrain-dominant CVR deficits in AD have been proposed as direct indicators of vascular dysfunction while CBF decreases in temporal and parietal cortices have been attributed to atrophy-related lower metabolic demand.^11^ Our results could denote similar independent and synergistic contribution of CVR deficits to FTD disease development.

Finally, despite the moderate strength of some discovered effects, RSFA demonstrated consistency across different analytical approaches and covariates of no interest. This is noteworthy, given increasing reproducibility concerns across analytical approaches in neuroimaging. While voxel-based analysis enables straightforward comparisons of statistical maps in clearly defined anatomical regions, ICA reduces multiple comparison burden and helps identify brain activation patterns that may be driven by different participants. The convergence of results across approaches and statistical models enhances the reliability of our results, providing directions for further mechanistic understanding and hypothesis-driven studies.

As a secondary objective, we examined the behavioral relevance of RSFA and found a relationship between RSFA reductions in sequence variation carriers and diminished global cognitive function. This accords with previous reports in AD^10^ and hemodynamic impairment.^23^ Higher RSFA in the PFC correlated with better global cognition, captured by PC 1, especially in symptomatic carriers, even after adjusting for age and disease progression effects. The behavioral significance of RSFA in the PFC was further highlighted using independent measures of executive function (eTable 8), consistent with previously documented relationships between structural and CBF changes and executive function in genetic FTD,^4^ including GENFI.^3,30^ These findings align with evidence from aging and FTD, showing increased dependence of successful cognition on precisely regulated large-scale brain networks.^51^ Furthermore, in the GENFI sample, stronger function-cognition coupling is described in presymptomatic carriers approaching their expected age at disease onset, in the absence of cognitive performance differences relative to noncarriers.^51^ Our observations support these findings and suggest that CVR may benefit cognition in individuals at FTD risk.

Several methodological remarks warrant consideration. First, the cross-sectional design precludes causal inferences, which necessitate longitudinal examination. Second, several uncovered effects only approached statistical significance, suggesting that the FDR correction was conservative, noting that interaction-moderation effects require large samples. Despite that, the RSFA effects in presymptomatic carriers resembled those of symptomatic cases, illustrating the vulnerability of the middle frontal and posterior cortices across 2 different analytical approaches. Similarly, the lack of effects in sequence variation carriers stratified by mutation does not rule out complex nonlinear relationships potentially obscured by insufficient power. As regression analyses assume linear relationships, nonlinear RSFA differences across variant groups or nonlinear age-related differences between controls and symptomatic carriers may have been overlooked. Future studies should test specific hypotheses about the role of cerebrovascular impairment in particular sequence variants (i.e., whether it directly contributes to neuropathology or is a general modifier across variants) and respective relationships to cognition, ensuring sufficient power and targeted analyses. These limitations underscore the need for larger, longitudinal FTD cohorts with diverse neuroimaging measures and nonlinear or machine learning modeling to elucidate gene-specific effects and genetic moderators across FTD subtypes and other dementias. Third, the delay between RSFA acquisition and cognitive assessment varied because of ongoing recruitment within GENFI, missing data for some participants, and heterogeneity in completed visits. The RSFA-cognition results remained unchanged after adjusting for differences between resting-state scan and cognitive testing, and the used PCA and robust regression are well-suited to handle missing values and outliers. However, discrepancies between the assessments may have hindered the sensitivity of our brain-cognition analyses. Finally, although RSFA-CVR offers an effective way to quantify resting BOLD signal variability noninvasively, RSFA may be attributed to other sources than vascular contractility, such as ion dynamics and cardiopulmonary fluctuations.^17,26^ Among frequency-domain methods, such as amplitude of low-frequency fluctuations (ALFFs) and fractional ALFF,^52^ fALFF demonstrates a weaker relationship with CO_2_-induced BOLD signal change than ALFF,^53^ implying that the frequency range may be critical for capturing vascular contributions. In addition, RSFA may reflect CBF effects, WM hyperintensities (WMHs), and cardiovascular factors.^26^ Alternative CVR mapping techniques, such as intermittent breath modulation,^12^ which does not require gas inhalation and offers higher sensitivity than RSFA-CVR, especially for noisy CVR data,^13^ could help clarify the vascular factors driving the reported RSFA changes. Other means to quantify cerebrovascular function include resting arterial-spin labeling–CBF and WMH burden on MRI. Future CVR investigations could incorporate such estimates, and CSF and blood markers, in relation to cognitive decline^22^ in a multimodal manner.^24,47^ At the clinical level, integrative approaches to uncover protective factors in prodromal stages of disease may improve prognosis and inform stratification, future trials, patients, and carers.

Using the RSFA approach, we found CVR alterations in presymptomatic and symptomatic FTD with a frontal and posterior cortical predilection, concordant across component-based and voxel-level analyses. We also showed that higher CVR yields a cognitive benefit, especially in individuals at elevated FTD risk. Our results suggest RSFA as a safe, tolerable, and clinically informative signal that may aid cerebrovascular health quantification in large-scale population studies among frail participants. We propose that there is a vascular contribution that interacts with FTD pathology in driving disease development. Cerebrovascular health may be a potential target for biomarker identification and a modifiable factor against clinical deterioration in people at genetic risk of FTD.

## Appendix Coinvestigators

**Table:**

Coinvestigators are listed at Neurology.org.

## Glossary

AD: Alzheimer disease
ALFF: amplitude of low-frequency fluctuation
BBB: blood-brain barrier
BOLD: blood oxygenation–level dependent
*C9orf72*: chromosome 9 open reading frame 72
CBF: cerebral blood flow
CVR: cerebrovascular reactivity
EPI: echo-planar imaging
FDR: false discovery rate
FTD: frontotemporal dementia
FTLD: frontotemporal lobar degeneration
GENFI: Genetic FTD Initiative
GM: gray matter
*GRN*: progranulin
ICA: independent component analysis
*MAPT*: microtubule-associated protein tau
MDL: minimum description length
MFG: middle frontal gyrus
MLR: multiple linear regression
MNI: Montreal Neurological Institute
PCA: principal component analysis
PCC: posterior cingulate cortex
PFC: prefrontal cortex
ROI: region of interest
RSFA: resting-state fluctuation amplitude
SFG: superior frontal gyrus
SPM: Statistical Parametric Mapping
TE: echo time
TFCE: threshold-free cluster enhancement
TR: repetition time
WM: white matter
WMH: WM hyperintensity
